# Multiple-Drugs-Related Osteonecrosis of the Jaw in a Patient Affected by Multiple Myeloma: A Case Report

**DOI:** 10.3390/dj11040104

**Published:** 2023-04-13

**Authors:** Mario Caggiano, Federica Di Spirito, Alfonso Acerra, Marzio Galdi, Laura Sisalli

**Affiliations:** Department of Medicine, Surgery and Dentistry “Scuola Medica Salernitana”, University of Salerno, Via Allende, 84081 Baronissi, Italy

**Keywords:** oral cavity, oral lesion, lenalidomide, adverse event, osteonecrosis of the jaws, adverse drug reaction

## Abstract

A 60-year-old woman suffering from multiple myeloma (MM) was treated with zoledronic acid (bisphosphonate), dexamethasone (corticosteroid), bortezomib (a chemotherapeutic agent), and lenalidomide (thalidomide analog) for about a year and with lenalidomide alone as maintenance therapy for almost two years and developed stage three medication-related osteonecrosis of the jaws (MRONJ) in the upper left dental arch approximately two weeks after tooth extraction, which was treated with a medical nonoperative conservative approach until reversion to stage one. The present case report describing the development of multi-drug-related osteonecrosis of the jaws during the pharmacologic MM maintenance phase draws attention to the complex multidisciplinary and multistage management of MM subjects and also that during disease remission, crucially involving oral healthcare providers for MRONJ prevention and pharmacovigilance. To prevent similar cases, cancer patient management should ensure proper dental care not only before starting but also throughout therapy duration and ensure continuous interdisciplinary consensus between oncologists and dentists. Moreover, also considering the independent negative and potentially synergistic effect on bone metabolism and mucosal healing processes of employed medicaments, additionally combined with the cumulative one of previous intravenous bisphosphonates, further studies should highlight the polypharmacy effect and hopefully aid in patient-specific MRONJ risk assessment in cancer patients.

## 1. Introduction

Medication-related osteonecrosis of the jaws (MRONJ) is a drug-related, adverse event characterized by progressive destruction and necrosis of the mandibular and/or maxillary bone in patients treated with drugs identified to be at an increased risk of MRONJ that has been ascertained without prior radiation treatment [1]. Antiresorptive (e.g., bisphosphonates, denosumab) and antiangiogenic drugs (e.g., anti-VEGF, TKIs, mTOR inhibitors) are the medications most commonly implicated in the development of MRONJ because of the impairment of the bone metabolism they cause [2]. Such an adverse event, initially described as bisphosphonate-related osteonecrosis of the jaw (BRONJ), was later referred to as drug-related osteonecrosis of the jaw (DRONJ), thus encompassing all osteonecrotic processes associated with drug use regardless of their mechanism of action [3], as evidence is accumulating for other drugs as well. Indeed, after bisphosphonates, several other drugs have been definitively or potentially implicated in this drug-related adverse event over time, and many more may yet be described as causing, predisposing, or triggering MRONJ so that the role of the clinicians in pharmacovigilance should be considered more important than ever.

These drugs are also used to treat multiple myeloma (MM), a malignant neoplasm characterized by clonal proliferation of plasma cells primarily harvested in the bone marrow. MM most frequently affects people after the age of 40, accounts for 1–1.8% of all neoplasms overall, and is the second most common hematologic malignancy [4]. MM diagnosis is made by evaluating bone marrow clonal plasma cells and M or monoclonal proteins in urine, serum, or both. Renal dysfunction, anemia, infection, bone pain, and osteolytic lesions with slow, progressive, and steady bone damage possibly leading to long bone fractures and spinal impairment are the clinical expression of the involvement of related organs and tissues characterizing MM symptomatic forms [5]. Bisphosphonate-related osteonecrosis of the jaw has long been documented in MM subjects with an estimated incidence of 5 to 10%, and its development is associated with the alteration of bone metabolism and inhibition of angiogenesis [6]. Other drugs administered in the treatment of MM, including bortezomib, thalidomide, and its analogs, also appear to increase the risk of MRONJ when administrated in conjunction with bisphosphonates [7].

The present case report describes the development of multi-drug-related ONJ in a 60-year-old woman with MM in the pharmacological maintenance phase with lenalidomide, a thalidomide analog with antiangiogenic properties and a direct cytotoxic effect, after MM initial therapy with dexamethasone, bortezomib, and zoledronic acid. The described case aimed to highlight the need for continuous and appropriate oral care in patients with multiple myeloma even after initial therapy both for the prevention and timely detection of MRONJ onset and to emphasize the need for a deeper understanding of the potential combined adverse effects of bisphosphonates and lenalidomide in MM subjects and the critical role of clinicians in pharmacovigilance.

## 2. Case Report

In February 2022, a 60-year-old woman who was an inpatient at the Department of Otorhinolaryngology of the San Giovanni di Dio and Ruggi d’Aragona University Hospital of Salerno* was referred to the Dental Complex Operating Unit of the hospital for specialist consultation. The patient, under Piperacillin 4.5 g, 1 vial 3 times a day, prescribed by otorhinolaryngologists, reported frequent loss of fluid from the nose and pain in the upper left dental arch for about 1 month.

**Figure 1:**
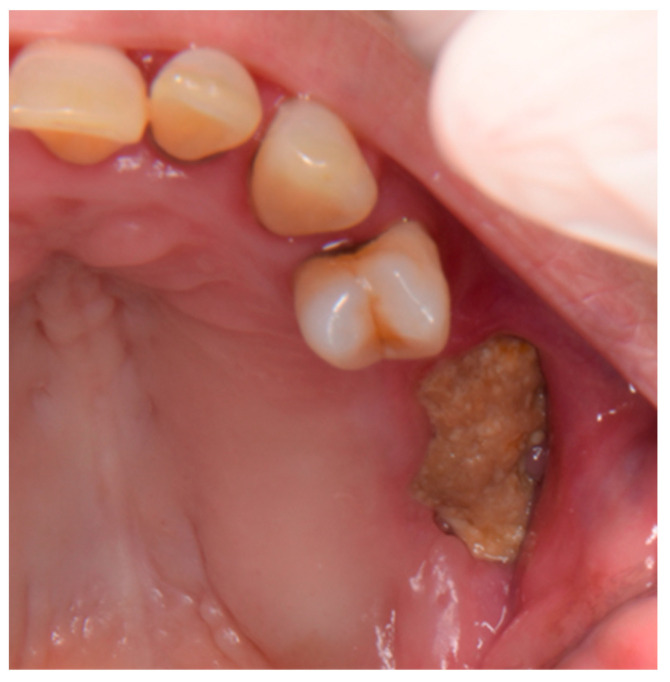
Exposed bone in the posterior area of the left upper jaw.

When the patient came for observation, the intraoral examination revealed swelling of the left posterosuperior gingiva, profuse spontaneous suppuration, and exposed bone in the posterior area of the left upper jaw (Figure 1).

A history of MM, diagnosed in May 2016, was recorded. In detail, MM was treated with lenalidomide, dexamethasone, bortezomib, and zoledronic acid for 18 months. After MM’s remission, MM maintenance therapy with lenalidomide alone was started in October 2020 because the patient was positive for CM IgG-Lambda at a low concentration by immunofixation. MM remained stable over time, and in September 2021, the patient tested positive for SARS-CoV-2 infection as an unvaccinated person with the febrile syndrome. However, no COVID-19-related oral lesions were detected in the oral cavity of the patient.

In addition, the patient reported a tooth extraction in the left posterior upper jaw 15 days before the onset of the described symptoms.

**Figure 2:**
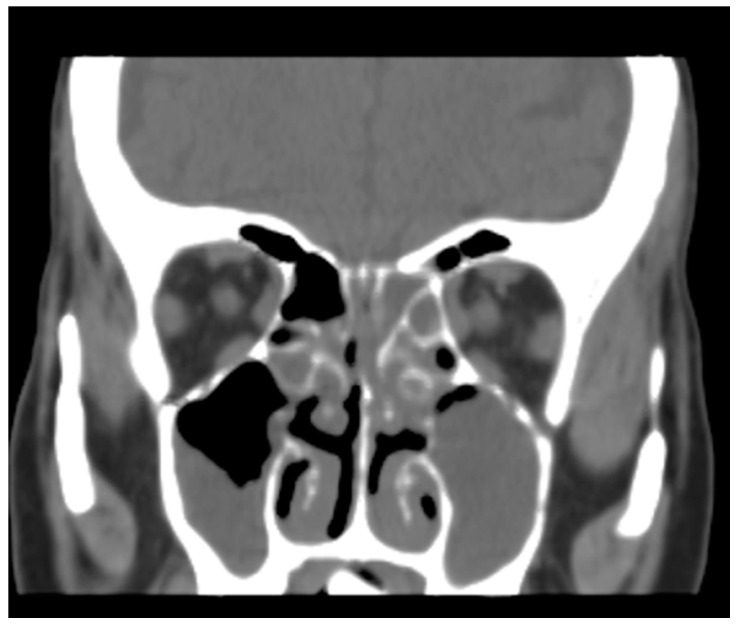
Bilateral obstruction of maxillary sinuses with no evidence of erosive changes in bony structures.

A CT scan of the paranasal sinus performed on the recommendation of otorhinolaryngologists in March 2017 was also obtained. The three-dimensional radiological exam showed a bilateral inflammation of the maxillary sinuses without erosive changes to the bony structures in the left upper jaw (Figure 2), thus excluding previous involvement of the area.

The patient, diagnosed with stage 3 MRONJ, was treated with metronidazole 250 mg, 1 tablet every 8 h (for 10 days), in combination with amoxicillin and clavulanic acid 1 g, 1 tablet every 12 h (for 14 days), and chlorhexidine 0.2%, twice daily, to resolve the local infection and conservatively reduce the severity of the oral adverse event [8,9]. No additional therapies were decided by interdisciplinary consensus [10].

**Figure 3:**
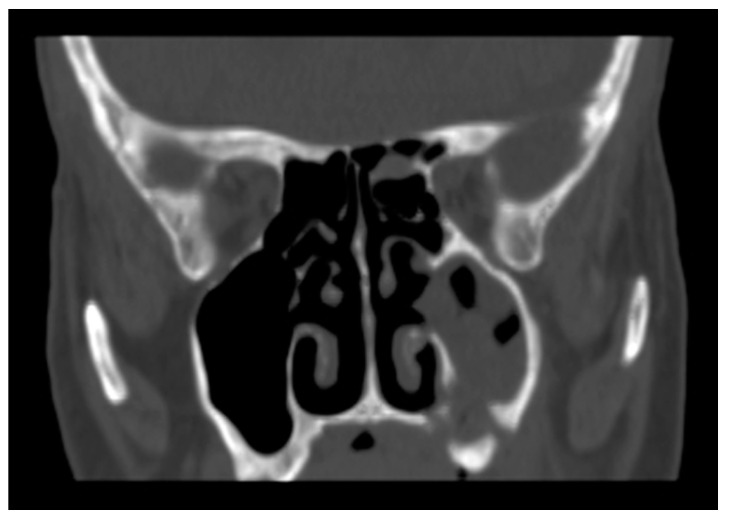
Complete obstruction of the left maxillary sinus with the erosion of the medial wall and floor of the sinus.

A new CT scan of the maxillary jaws was requested and performed in March 2022. The images showed disruption of the alveolar bone in the upper left dental arch with oro-antral communication, solution of continuity with the lateral wall of the nasal fossa, complete obstruction of the left maxillary sinus with the erosion of the medial wall and sinus floor, and involvement of the alveolar bone (Figure 3).

**Figure 4:**
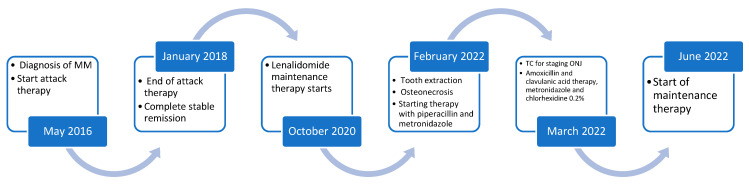
Patient’s medical history timeline.

Stage 1 MRONJ was successfully achieved with nonoperative medical conservative therapy with spontaneous suppuration and soft tissue inflammation resolution and asymptomatic, exposed bone free from local infection [11]. Subsequently, MRONJ maintenance therapy with chlorhexidine 0.1%, mouth rinsed twice daily, was performed until June 2022, waiting for the spontaneous demarcation and mobilization of bone sequestration prior to careful removal of the necrotic bone, which was assigned to the Maxillo-Facial Unit of the hospital (Figure 4).

## 3. Discussion

The present case report described the development of multi-drug-related ONJ in a 60-year-old female with MM in the pharmacological maintenance phase, aiming to highlight the need for continuous and appropriate oral care in patients with multiple myeloma even after initial therapy both for prevention and timely detection of MRONJ onset and to emphasize the need for a deeper understanding of the potential combined adverse effects on MRONJ genesis of bisphosphonates, chemotherapy, corticosteroids, and lenalidomide in MM subjects, directly involving clinicians in pharmacovigilance.

The presented case of multi-drug-related ONJ occurred in a female MM patient in agreement with previous findings, confirmed by recent age- and gender-based epidemiological studies comparing the occurrence of MRONJ following the administration of drugs with antiresorptive and antiangiogenic activities, showing that the female gender is significantly more frequently affected by this adverse event after therapy with antiresorptive drugs [12,13]. This evidence may be secondary to the higher prevalence of antiresorptive drug therapies in women compared to men in the context of postmenopausal osteoporosis. Indeed, exclusively considering cancer subjects under antiresorptive treatment, such a gender difference would no longer be apparent, as breast and prostate cancer are the two malignancies with the highest incidence in the two genders, respectively [14,15].

The most common clinical signs of MRONJ include exposed necrotic bone in the oral cavity, a history of pain (odontalgia, weight-bearing bone pain, myogenic pain, sinus pain, trigeminal-type pain), and disruption of local wound healing processes [16]. Accordingly, the patient reported pain in the upper left dental arch for approximately one month and presented with exposed bone in the posterior area of the left upper jaw. Other less common signs typically described comprise intra- and extraoral soft tissue swelling, intra- and extraoral fistula, nasal discharge, sinusitis ipsilateral to necrosis, purulent discharge, odontogenic abscesses, and halitosis, which is also consistent with the current case presenting with swelling of the left posterosuperior gingiva, abscess collection, and frequent fluid loss from the nose [16]. In the most severe cases, an extension of the necrotic process to the basal bone, pathologic fractures, or nerve impairment, especially of the mandible, may be noted, which were fortunately not found in the case illustrated [16]. In contrast to the anatomical risk factors, which generally rate the mandible as the most affected jaw in 75% of cases, the upper jaw was unilaterally affected in the presented case [17].

The reported case was initially diagnosed as stage 3 MRONJ, applying, among the classifications proposed [18], the one developed by Ruggiero et al. The last, based on cases’ pharmacological histories and MRONJ clinical and radiological features, guides MRONJ management [19,20,21], identifying stage 1: with asymptomatic exposed bone in the oral cavity and without adjacent soft tissue swelling or infection—at this stage, some patients may have pain before clinical and radiographic appearance; stage 2: with exposed bone and adjacent soft tissue swelling and secondary infection; stage 3: with signs similar to stage 2 but associated with more severe infection, being more difficult to be pharmacologically managed, and possibly exacerbated by extraoral fistulas, nasal discharge, and pathologic fractures [1,22]. Recently, a stage 0 was also introduced, which categorizes all MRONJ cases without clinical signs of necrotic bone but with non-periodontitis-related tooth loss, intraoral swelling, unjustified odontalgia, temporomandibular and sinus pain, neurosensory alterations, bone loss and/or sclerosis, and changes in the periodontal ligament space [23].

Clinical diagnosis was implemented by three-dimensional radiographic findings, as previously recommended, to improve the identification of the extent of bony involvement by evaluating bone changes and osteosclerosis and thus MRONJ staging and management [1,18,19,24].

Nonoperative therapy, which was the primary treatment chosen in the described case and successfully reverted stage 3 MRONJ to stage 1, has been recommended, similarly to the surgical approach at each stage to resolve earlier stages and stabilize and revert disease progression in severe ones [8,9,25]. Therefore, such a nonoperative medical conservative approach is considered curative while preserving or improving the quality of life and prioritizing the management of the oncologic condition [8,9]. Indeed, although many authors consider the conventional or laser-assisted surgical approach to be the treatment of first choice regardless of stage, including earlier ones aiming to remove necrotic bone, smoothen residual sharp bone edges, and obtain mucosal closure by primary intention, a conservative medical approach with topical antiseptics and systemically-delivered antibiotics is also considered effective in improving or maintaining the manifestation as asymptomatic in 75% of cases and is preferred in those subjects not eligible for surgery [23,25,26,27,28]. Moreover, conservative treatment in some cases may also benefit from the administration of tocopherol (vitamin E) in combination with pentoxifylline, as has been proposed for osteoradionecrosis [19,20]. However, tocopherol has not been currently prescribed because it was not considered sufficiently safe due to the patient’s general condition and polypharmacy. The surgical removal of the necrotic bone was assigned to the Maxillo-Facial Unit of the hospital after the successful regression to stage 1 and is currently deferred while awaiting spontaneous demarcation of the bone sequestrum. Additional therapeutic options also aiming at bone tissue repair and regeneration may be critical in treating severe MRONJ and subsequent management of bone healing, atrophy, and prosthetic rehabilitation [21,22,29,30].

Although the pathogenesis of drug-related adverse events is not fully understood, the process is considered multifactorial. Several drug-related mechanisms, mainly proposed for bisphosphonates and currently attributed to zoledronic acid administration, involving altered bone turnover due to inhibition of osteoclastic and osteoblastic activity, reduction in angiogenic activity with damage to the microcirculation, bone ischemia with subsequent avascular necrosis, congenital or acquired immune system dysregulation, and precipitating events, including chronic trauma and infectious–inflammatory foci, may be implied [19,31]. Multiple myeloma is among the diseases with a recognized increased risk of developing MRONJ, mainly related to bisphosphonates. In detail, the incidence of bisphosphonates-related osteonecrosis of the jaw has been estimated to range between 5 and 10% in MM subjects. It is thought to be related to adynamic bone and angiogenesis inhibition [6]. Therefore, appropriate oral care and dental hygiene and avoidance of unnecessary dental procedures are strongly advocated in MM subjects about to start and under bisphosphonates as a primary preventive strategy for bisphosphonates-related osteonecrosis of the jaw.

Moreover, bortezomib, thalidomide, and its analogs, such as lenalidomide and corticosteroids, used as part of MM treatment, increasing treatment responsiveness and duration, may also be involved somehow and to varying degrees in the development of MRONJ onset [6,7,30].

In detail, bortezomib is a chemotherapeutic agent that acts by inhibiting proteasomes. Although it does not directly increase the risk of venous thromboembolism, it may still exert antithrombotic effects by decreasing the expression of adhesion molecules on endothelial and vascular cells and inhibiting platelet aggregation. However, venous thromboembolism risk related to bortezomib, fortunately, seems to be very low (1%), besides the reported association with thrombocytopenia (20–26% and 4–17%); to date, no direct evidence of bortezomib’s role in MRONJ pathogenesis is available [32].

Lenalidomide (CC-5013: Revlimid) is a thalidomide-derived immunomodulator that is considered very effective (58–59%) in combination with dexamethasone in relapsed or refractory MM, although it is potentially related to adverse events [33,34,35]. The risk of venous thromboembolism associated with lenalidomide was estimated to be 0–≤5% when administered alone and increased to 8.5–15% when combined with dexamethasone, while the incidence of neutropenia and thrombocytopenia ranged from 28–59% and 17–62%, respectively [7]. Lenalidomide may also be responsible for myelosuppression and teratogenic effects and is, therefore, contraindicated in pregnancy. Skin rash, diarrhea, pruritus, anemia, neurologic toxicity, prothrombotic effects, renal failure, and asthenia occur in 31–36% of the cases, and rarely do patients develop pneumonia, hypothyroidism, or hypogonadism [7].

The development of MRONJ after drug administration is generally expected to be approximately 24 months for intravenous bisphosphonates, 36–48 months for oral bisphosphonates, and 9.75 months for antiangiogenic drugs, respectively. Hence, the currently recorded time to MRONJ onset after multiple drugs potentially related to the described manifestation appears to be shorter than the time usually recorded for bisphosphonates and antiangiogenic drugs. This suggests a cumulative exacerbating role of bortezomib, corticosteroids, and lenalidomide in MRONJ genesis, aggravating MRONJ risk associated with prior zoledronic acid administration.

Moreover, a definitive determination of drugs’ causative role in MRONJ genesis has been proposed to be considered aleatory conceivably, especially taking into account that the risk of developing MRONJ in cancer patients not treated with the incriminated drugs is still between 0 and 0.7%, so it should not be considered completely nonexistent [8,36]. However, several clinical studies have shown that MRONJ occurs mainly in patients treated with bisphosphonates, such as zoledronate (3.2% at 3 years).

Furthermore, 50% of MRONJ cases described after tooth extractions were declared to be associated with preexisting odontogenic infections/inflammations, biasing estimates of overall developmental risk and case incidence even though the patient had not previously reported symptoms of local infection/inflammation [37]. However, the potential precipitating or predisposing effect of dental extractions was previously reported to range from 1.6 to 14.8% in cancer patients and remains controversial [38,39,40].

Therefore, considering the potential cumulative pathogenic effect resulting from the use of multiple drugs, corticosteroids, and bortezomib, potentially promoting and determining the development of MRONJ and derived from the previously administered zoledronic acid, the exact precipitating or predisposing role of each drug in MM could not be determined with certainty, thus constituting the main limitation of this study.

Consequently, future studies are needed to evaluate the potential synergistic exacerbating effects of MM combined pharmacologic treatments with bisphosphonates, antiangiogenetic drugs, corticosteroids, chemotherapeutic agents, and thalidomide analogs [41,42].

## 4. Conclusions

The present case report described the development of multi-drug-related ONJ in a 60-year-old woman with multiple myeloma in the pharmacologic maintenance phase, potentially due to the cumulative exacerbating pathogenic effect of the use of lenalidomide, corticosteroids, and bortezomib after zoledronic acid administration.

The presented case of multiple-drugs-related ONJ development during the MM pharmacological maintenance phase may further draw attention to the complex multidisciplinary and multistage management of MM patients and also that during disease remission and maintenance therapy, involving oral healthcare providers for MRONJ prevention and pharmacovigilance. In detail, to prevent similar cases, the management of cancer patients should ensure appropriate dental care not only before initiation but also throughout the duration of therapy and ensure continuous interdisciplinary consensus between oncologists and dentists. Increased pharmacovigilance against all new drugs that may affect bone turnover either by direct action on osteoclasts or by indirect action on bone blood flow should be strongly advocated.

Moreover, further studies should shed light on the independent negative effect on bone metabolism and mucosal healing processes exerted and the potential synergistic pathogenic effect of these medicaments additionally combined with the cumulative one of intravenous bisphosphonates; further studies should highlight the effect of polypharmacy on bone metabolism in cancer patients and hopefully aid in patient-specific MRONJ risk estimates.

In the meantime, according to the current evidence-based recommendations, oral healthcare providers should, as a primary prevention strategy, consider all subjects currently or previously under antiresorptive and antiangiogenetic drugs at higher risk to develop MRONJ compared with the general population, especially if suffering from cancer rather than osteo-metabolic diseases and are being treated with or have taken zoledronic acid and denosumab in the past. The management of MRONJ, particularly in neoplastic patients, including those with MM, should be discussed in detail with the multidisciplinary team, evaluating the case-specific risk–benefit ratio and appropriate treatment planning.

## Data Availability

All data generated or analyzed during this study are included in this article.
